# Report of two siblings with spondylodysplastic Ehlers-Danlos syndrome and B4GALT7 deficiency

**DOI:** 10.1186/s12887-021-02767-0

**Published:** 2021-06-30

**Authors:** Delia Lorenz, Wolfram Kress, Ann-Kathrin Zaum, Christian P. Speer, Helge Hebestreit

**Affiliations:** 1 Center for Rare Diseases, University Hospital Würzburg, University of Würzburg, Josef-Schneider-Strasse 2, 97080 Würzburg, Germany; 2 University Children’s Hospital Würzburg, University of Würzburg, Josef-Schneider-Strasse 2, 97080 Würzburg, Germany; 3 Department of Human Genetics, University of Würzburg, Biozentrum, Am Hubland, 97074 Würzburg, Germany

**Keywords:** Connective tissue disorder, Spondylodysplastic Ehlers-Danlos syndrome, *B4GALT7* gene, Case report

## Abstract

**Background:**

The spondylodysplastic Ehlers-Danlos subtype (OMIM **#**130070) is a rare connective tissue disorder characterized by a combination of connective tissue symptoms, skeletal features and short stature. It is caused by variants in genes encoding for enzymes involved in the proteoglycan biosynthesis or for a zinc transporter.

**Presentation of cases:**

We report two brothers with a similar phenotype of short stature, joint hypermobility, distinct craniofacial features, developmental delay and severe hypermetropia indicative for a spondylodysplastic Ehlers-Danlos subtype. One also suffered from a recurrent pneumothorax. Gene panel analysis identified two compound heterozygous variants in the *B4GALT7* gene: c.641G > A and c.723 + 4A > G. *B4GALT7* encodes for galactosyltransferase I, which is required for the initiation of glycosaminoglycan side chain synthesis of proteoglycans.

**Conclusions:**

This is a first full report on two cases with spondylodysplastic Ehlers-Danlos syndrome and the c.723 + 4A > G variant of *B4GALT7.* The recurrent pneumothoraces observed in one case expand the variable phenotype of the syndrome.

## Background

The Ehlers-Danlos syndromes (EDS) are a large heterogeneous group of heritable connective tissue disorders. The 2017 classification regrouped the EDS into six groups (A to F) according to underlying genetic and pathogenic mechanisms [1]. While the classical EDS is part of the disease group A in which collagen structure and collagen processing is affected resulting in the main clinical features of skin hyperextensibility and joint hypermobility, EDS entities of other genetic / pathogenic groups have a wider phenotypic variability.

The EDS spondylodysplastic type (group D: disorders of glycosaminoglycan biosynthesis) is either caused by homozygous or compound heterozygous mutations in the *B4GALT7* gene on chromosome 5q35, or by mutations in the *B3GALT6* gene (galactosytransferases) (OMIM **#** 615349) or in the *SLC39A13* gene (zinc transporter) (OMIM **#** 612350) [2, 3]. It is inherited in an autosomal recessive pattern. The phenotype is defined by short stature and generalized muscular hypotonia as major criteria, and skin hyperextensibility, soft doughy skin, pes planus, delayed motor and cognitive development and osteopenia as minor criteria. There are also gene-specific minor criteria such as severe hypermetropia, characteristic craniofacial features, bilateral elbow contractures and radiographic findings, e.g. radioulnar synostosis for *B4GALT7* enzyme deficiency.

The *B4GALT7* protein is a member of the beta-1,4-galactosyltransferase family and is involved in the formation of the glycosaminoglycan-protein bond in proteoglycans. The enzyme encoded by this gene attaches the first galactose in the common carbohydrate-protein linkage found in proteoglycans. Defective structures or deficient quantities of this enzyme lead to insufficient amounts of mature functional proteoglycans [4, 5] .

We describe two siblings who are compound heterozygous for variants in the *B4GALT7* gene – the splice variant (c.723 + 4A > G) described provisionally [6] and the missense variant previously reported in mutation databases (e.g. HGMD: CM166199, ClinVar: RCV000239469.1604327.007, OMIM #604327.007) - further confirming the variable phenotypic spectrum associated with pathogenic variants in this gene.

## Presentation of cases

The two brothers (18 and 15y) were patients in our neuropediatric department since 2003 and were later seen in the Center for Rare Diseases. The parents and patients gave consent for genetic analysis and publication.

Clinical description.

Patient 1 (male, 18 years): Pregnancy and delivery at term were normal, as were the anthropometric measures at birth (Table 1). Hypothyroidism was diagnosed at the age of 6 months and severe hypermetropia at the age of 3 years. Additional symptoms reported at the age of 5.4 years were delayed (fine) motor development and short stature. Clinical follow up at 9.4 years revealed delayed cognitive development, flat, skew feet and a hoarse voice. From the age of 15 to 17 years, the patient suffered from recurrent spontaneous pneumothoraces that were finally treated by pleurectomy and pleurodesis. Furthermore, he presented with several patellar luxations. Clinical examination at the age of 18 years showed short stature with a relatively large head, joint hypermobility, and distinct craniofacial features (big eyes, thin lips and small mouth, deep set ears).

**Table 1:** Summary of patients’ symptoms

		Patient 1 (18 years)	Patient 2 (15 years)
*Gestational age*		40 weeks	41 weeks
*Anthropometry at birth*	Height	48 cm (3rd centile)	51 cm (10th centile)
	Weight	3310 g (25th centile)	3160 g (10th to 25th centile)
*Anthropometry*	Height	156.5 cm (3 cm <3rd centile)	148.5 cm (6 cm <3rd centile)
	Weight	68.2 kg (50th centile)	49.2 kg (10. centile)
	Head circumference	59 cm (90th centile)	57.5 cm (75th to 90th centile)
*Development*	Motor	Delayed	Delayed
	Speech	–	Delayed
	Cognitive	Delayed	
*Craniofacial features*		Big eyes, thin lips, deep set ears	Thin lips, prominent forehead
*Musculoscelettal*		Joint hypermobility	Joint hypermobility
		Flat, skew feet	Flat, skew feet, crossing of 2nd and 3rd toe
			Scoliosis
*Eyes*		Severe hypermetropia	Severe hypermetropia
*Other*		Hypothyroidism	Hypothyroidism
		Recurrent pneumothoraces	
		Patellar luxations	
		Hoarse voice	

Patient 2 (male, 15 years): No complications occurred during pregnancy and delivery, length and weight at birth were normal (Table 1). Hypothyroidism was diagnosed at the age of 4 months. The boy was seen again at the age of 2.2 years, when he presented with developmental delay (free walking started at the age of 2.1 years, word pool of approximately 20 words), short stature, and severe hypermetropia. At the age of 5.0 years and 6.3 years, additional diagnoses comprised nutritional problems, speech developmental disorder, and flat, skew feet. Clinical examination at age 15 years showed persistent short stature and low weight, scoliosis, joint hypermobility, distinct craniofacial features (small lips, prominent forehead), and crossing of 2nd and 3rd toes.

The non-consanguineous parents came from Romania. There was no family history for short stature, joint hyperextensibility, skin laxity or dysmorphic features.

## Radiographic features

**Fig. 1:**
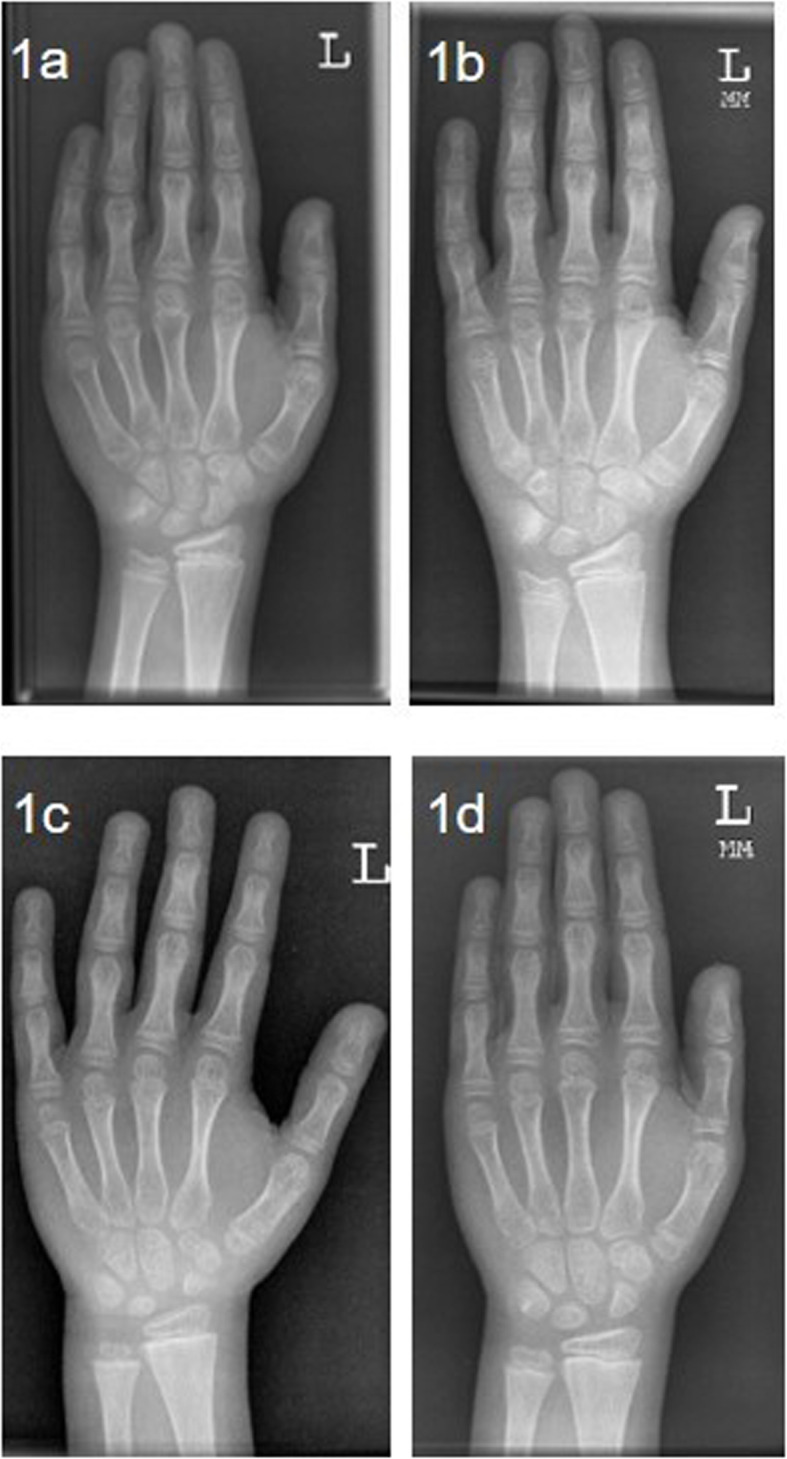
**a** Patient 1: Hand and wrist radiography 9y: accelerated bone development approx. 11y; **b** Patient 1: Hand and wrist radiography 10y 10 m: accelerated bone development approx. 12y 6 m; **c** Patient 2: Hand and wrist radiography 5y 9 m: slightly accelerated bone development approx. 7y; **d** Patient 2: Hand and wrist radiography 7y 8 m: slightly accelerated bone development approx. 9y

**Fig. 2:**
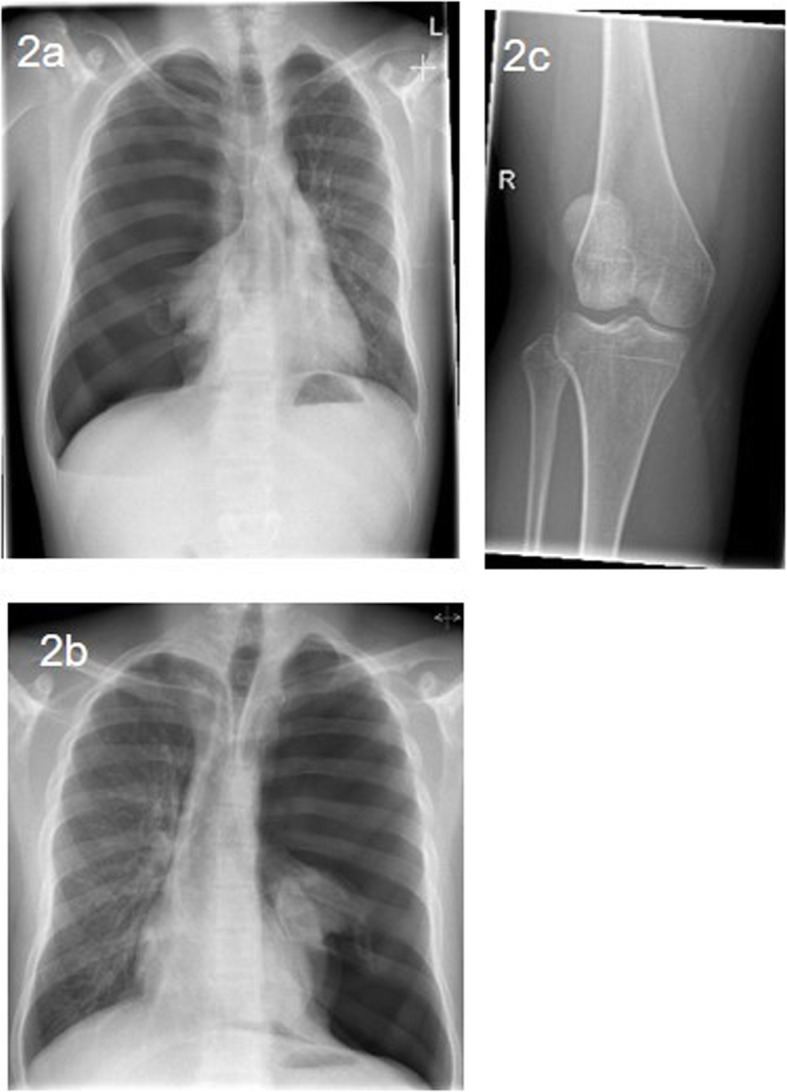
**a** Patient 1: chest x-ray, first tension seropneumothorax right side with mediastinal shift, 15y; **b** Patient 1: chest X-ray, tension pneumothorax left side with mediastinal shift, 16y; **c** Patient 1: X-ray right knee because of patellar luxation, no osteopenia, 20y

Within the diagnostic work-up for small stature, hand and wrist radiographs were taken in both brothers. They revealed an accelerated bone development of approximately 2y in patient 1 and a slightly accelerated bone development in patient 2 (Fig. 1a–d). Due to recurrent pneumothoraces, 37 chest X-rays (Fig. 2a, b) were taken of patient 1, and one X-ray of the right knee because of recurrent patellar luxations (Fig. 2c). X-rays showed no signs of osteopenia.

## Genetic analysis

Previous genetic evaluation in patient 1 included a chromosomal analysis at age 6 years and an array-CGH at age 14 years with normal results. An Ehlers-Danlos syndrome with its various subtypes was considered. Therefore, NGS panel analysis (TruSight One (Illumina, San Diego, CA USA)) was done for both brothers to identify their shared disease-causing variants. Targeted enrichment was performed by Nextera Rapid Capture from genomic DNA according to manufacturer’s protocols and sequencing was done on a NextSeq500 desktop sequencer (Illumina). Sequence coverage was at average 123x for the analyzed genes. Raw data (fastQ) were aligned to the human reference genome (GRCh37) using an integrated aligner in GensearchNGS (PhenoSystems SA, Wallonia, Belgium). This software was also applied for variant calling, visualization and filtering of variants. Variants were filtered according to the following criteria: variant frequency greater than 25%, minor allele frequency below 1%, occurrence in both brothers. Classification of variants was performed according to American College of Medical Genetics and Genomics **(**ACMG) guidelines [7] using Alamut Visual (Interactive Biosoftware, Rouen, France) including incorporated tools and databases. Sanger sequencing was done using routine protocols on an ABI3730 sequencing machine (Applied Biosystems, Foster City, USA).

## Results

Gene panel analysis revealed compound heterozygous variants in the *B4GALT7* gene, c.723 + 4A > G in Intron 4 and c.641G > A in exon 4 in both brothers. The parents were each heterozygous for one of the variants identified. The c.723 + 4A > G variant was inherited from the mother and the c.641G > A variant was inherited from the father. The variant c.641G > A (p.C214Y) is a known disease-causing variant that has been published before in a patient with EDS in compound heterozygous state with a known pathogenic variant (PM3, PP5 according to ACMG criteria) [8]. Additionally, the variant is very rare as annotated in gnomAD browser [9] (PM2), predicted to be pathogenic by in silico tools (e.g. PolyPhen2 [10], MutationTaster [11] (PP3)) and was found in the catalytic domain of the protein (PM1). Therefore, the variant was classified as likely pathogenic according to ACMG guidelines. The splice variant c.723 + 4A > G has been described once before in a presentation at the Variant Effect Prediction Training Course (VEPTC) [6], showing functionally alternative splicing on mRNA level. The variant was detected in trans to a pathogenic variant (this case) (PM3), it is very rare (not detected in population databases according to gnomAD [9]) (PM2), prediction algorithms for splicing (Alamut program package) were in favor of a functional effect (PP3). For these reasons, the variant was classified as a variant of uncertain significance according to the ACMG guidelines, with a strong indication towards a pathogenic variant, as it is seen in both affected brothers (and unpublished functional analysis indicate alternative splicing [6]).

## Discussion and conclusions

We report two brothers with genetically confirmed spondylodysplastic EDS associated with bi-allelic heterozygous pathogenic variants, a well-known c.641G > A variant and variant c.723 + 4A > G reported only provisionally before [6]. Our findings demonstrate that recurrent pneumothoraces occur in *B4GALT7*-related spondylodysplastic EDS, thereby expanding the spectrum of clinical presentations.

Clinical features of our patients were besides the major criteria short stature and mild muscular hypotonia, distinct craniofacial features, a developmental delay, and the already described early onset severe hypermetropia [8, 12, 13]. The older brother furthermore had recurrent pneumothoraces, which have not been described in other patients within the spondylodysplastic EDS spectrum and which reoccurred despite pleurodesis. The single other patient compound heterozygous with the c.641G > A variant reported so far, a 3.5 years old male, showed similar clinical symptoms: short stature, hypermobility, hyperelastic skin, as well as motor and cognitive delay [8]. Both of our patients received the diagnosis of hypothyroidism in infancy. Endocrinological manifestations have not been associated with spondylodysplastic EDS so far. Thus, we cannot relate the diagnosis of hypothyroidism with the syndrome in our cases, but also cannot exclude a connection.

Thirty-six patients from 31 families with molecularly confirmed spondylodysplastic EDS due to *B4GALT7*-deficiency with a total of 13 different variants have been reported so far (Table 2) [8, 12–24]. The likely pathogenic c.641G > A variant has been described for the first and only time in 2016 in a single patient [8]. Although the c.723 + 4A > G variant was strictly classified as variant of uncertain significance, the fact that it has been mentioned once in a presentation at the Variant Effect Prediction Training Course (VEPTC) and that it was detected in both brothers with the same clinical phenotype and only heterozygously in a parent strongly indicates a pathogenic effect of the variant. Therefore, our cases present a novel c.723 + 4A > G splice variant that very likely has phenotypical consequences.

**Table 2:** Patients with *B4GALT7* variants: review of the literature

		Hernandez et al. (1979, 1981, 1986) [22–24]	Kresse et al. (1987) [14]	Faiyaz-Ul-Haque et al. (2004) [15]	Guo et al. (2013) [16]	Cartault et al. (2015) [18]	Arunrut et al. (2016) [13]	Salter et al. (2016) [8]	Ritelli et al. (2017) [12]	Sandler-Wilson et al. (2019) [19]	Mihalic Mosher et al. (2019) [17]	Caraffi et al. (2019) [20]	This study	Total (patients with mutations)
Genetics	B4GALT7 variants	?	c.557C > A c.617 T > A	c.808C > T c.808C > T	c.122 T > C c.808C > T	c.808C > T c.808C > T	c.970 T > A c.970 T > A	c.277dupC c.641G > A; c.421C > T c.808C > T	c.829G > T c.829G > T	c.421C > T c.808C > T	c.398A > G c.808C > T	c.277_278insC c.628C > T	c.723 + 4A > G c.641G > A	26/36 homozygous 10/36 compound heterozygous
	Variants on protein	?	p.A186D p.L206P	p.R270C p.R270C	p.L41P p.R270C	p.R270C p.R270C	p.C324S p.C324S	p.H93Pfs*73 p.C214Y; p.R141W p.R270C	p.E277* p.E277*	p.R141W p.R270C	p.Q133R p.R270C	p.H93Pfs*73 p.H210Y	splicing p.C214Y	
Gender	Male	5	1	1	1	11		1		1	1	1	2	20
	Female			1		11	1	1	1	1				16
Age		8,15,15, 16,18y	4y 9 m	2, 33y	10y	4 to 46y	5y	3y 6 m, 13y	30y	4,10y	1d†	7y 8 m	15, 18Y	

Table modified from [20]

The majority of the 13 reported variants associated with spondylodysplastric EDS are localized in the large C-terminal catalytic domain [8, 20], whereas one variant (p.L41P) is localized in the transmembrane domain [16]. The most frequently identified variant is p.R270C. The p.R270 variant was reported in 22 homozygous patients from the same ethnic group (“white creoles”) with Larsen La Reunion syndrome (LRS) [18]. The LRS is more of a skeletal dysplasia phenotype including multiple joint dislocations with ligamentous hyperlaxity and a frequent radioulnar synostosis, as well as severe short stature and distinct facial features [18, 22]. Other studies described varying phenotypes in patients with compound heterozygosity or homozygosity for the p.R270C variant [8, 15–17, 19]. Biochemical studies showed a reduced galactosyltransferase activity in patients with p.R270C and p.A186D variants, whereas other variants (p.L206P and p.Q133R) lead to complete loss of enzyme function [4, 5], and correspondingly to severely affected or perinatal lethal phenotypes [14, 17]. The varying phenotypic spectrum within the B4GALT7 related spondylodysplastic EDS, even in patients with the same variant, is not fully understood. Explanations may include fluctuating quantitative effects on glycosaminoglycan biosynthesis, interactions with other variants in close linkage disequilibrium to B4GALT7 and / or the involvement of modifier genes.

The findings in this family expand the clinical phenotype of *B4GALT7*-spondylodysplastic EDS and provides evidence for the pathogenetic role of the c.723 + 4A > G splice variant. Within the group of patients with a combination of short stature, skeletal and connective tissue abnormalities, next generation sequencing can help to identify the underlying genetic cause.

## Data Availability

The datasets used and/or analysed during the current study are available from the corresponding author on reasonable request.

## Abbreviations

EDS: Ehlers-Danlos syndrome
LRS: Larson La Reunion syndrome
